# Large‐effect mutations generate trade‐off between predatory and locomotor ability during arms race coevolution with deadly prey

**DOI:** 10.1002/evl3.76

**Published:** 2018-07-31

**Authors:** Michael T. J. Hague, Gabriela Toledo, Shana L. Geffeney, Charles T. Hanifin, Edmund D. Brodie, Edmund D. Brodie

**Affiliations:** ^1^ Department of Biology University of Virginia Charlottesville Virginia 22903; ^2^ Department of Biology Utah State University Uintah Basin Vernal Utah 84322; ^3^ Department of Biology Utah State University Logan Utah 84322

**Keywords:** Antagonistic pleiotropy, sodium channel (Na_V_1.4), trade‐offs, tetrodotoxin (TTX)

## Abstract

Adaptive evolution in response to one selective challenge may disrupt other important aspects of performance. Such evolutionary trade‐offs are predicted to arise in the process of local adaptation, but it is unclear if these phenotypic compromises result from the antagonistic effects of simple amino acid substitutions. We tested for trade‐offs associated with beneficial mutations that confer tetrodotoxin (TTX) resistance in the voltage‐gated sodium channel (Na_V_1.4) in skeletal muscle of the common garter snake (*Thamnophis sirtalis*). Separate lineages in California and the Pacific Northwest independently evolved TTX‐resistant changes to the pore of Na_V_1.4 as a result of arms race coevolution with toxic prey, newts of the genus *Taricha*. Snakes from the California lineage that were homozygous for an allele known to confer large increases in toxin resistance (Na_V_1.4^LVNV^) had significantly reduced crawl speed compared to individuals with the ancestral TTX‐sensitive channel. Heterologous expression of native snake Na_V_1.4 proteins demonstrated that the same Na_V_1.4^LVNV^ allele confers a dramatic increase in TTX resistance and a correlated decrease in overall channel excitability. Our results suggest the same mutations that accumulate during arms race coevolution and beneficially interfere with toxin‐binding also cause changes in electrophysiological function of the channel that may affect organismal performance. This trade‐off was only evident in the predator lineage where coevolution has led to the most extreme resistance phenotype, determined by four critical amino acid substitutions. If these biophysical changes also translate to a fitness cost—for example, through the inability of *T. sirtalis* to quickly escape predators—then pleiotropy at this single locus could contribute to observed variation in levels of TTX resistance across the mosaic landscape of coevolution.

**Impact Summary**

Evolutionary trade‐offs are commonly expected to arise during the process of adaptation. As populations diverge and adapt to local conditions, compromises can develop between related traits, like virulence and spore production in pathogens, or microbial resistance and growth in plants. At a mechanistic level, it is almost entirely unclear how genetic changes mediate these higher level ecological trade‐offs. This study bridges that gap by linking specific mutations that evolved in response to one selective challenge, deadly prey, to consequences for protein function and organismal performance that present an ecological cost. Garter snakes in western North America evolved TTX resistance as a result of arms race coevolution with their toxic prey, newts of the genus *Taricha*. We found that trade‐offs at multiple levels of biological organization occur due to beneficial mutations that confer tetrodotoxin (TTX) resistance in the skeletal muscle sodium channel (Na_V_1.4) of the common garter snake (*Thamnophis sirtalis*). Snakes with toxin‐resistant mutations in Na_V_1.4 had significantly reduced crawl speed, a whole‐animal measure of muscle performance. The same set of TTX‐resistant mutations reduces the overall excitability of voltage‐gated sodium channels, a critical component of the vertebrate nervous system. These results suggest that the antagonistic effects of just a small number of amino acid substitutions at a single locus have the potential to influence broader ecological trade‐offs and drive mosaic patterns of adaptation across the landscape.

The evolutionary process occurs in the context of economic limitations, such that adaptations in one phenotypic dimension can compromise structure or function in another (Lynch and Gabriel 1987; Whitlock 1996; Brodie and Brodie 1999; Ghalambor et al. 2004; Kawecki and Ebert 2004; Bono et al. 2017). Specialization for a specific task is predicted to reduce overall performance in others (i.e., a jack‐of‐all‐trades is a master of none; Huey and Hertz 1984; Thompson 1986, 1994; Futuyma and Moreno 1988; Remold 2012). Consequently, as populations diverge and adapt to local conditions, compromises can arise at the phenotypic level, for example, between virulence and spore production in pathogens (Thrall and Burdon 2003), or microbial resistance and growth in plants (Todesco et al. 2010). But, at an underlying molecular level, these trade‐offs must be driven to a degree by specific changes in protein function and biomechanics, such that the biophysical changes of a single mutation may be beneficial in one sense, but disruptive to other important aspects of performance (Wang et al. 2002; DePristo et al. 2005; Weinreich et al. 2006; Harms and Thornton 2013; Natarajan et al. 2016; Storz 2016). In this respect, pleiotropy is thought to be an important driver of broader phenotypic patterns of adaptation; however, a functional link between changes in the structure of individual proteins and population variation in phenotypic trade‐offs remains tenuous (Hall et al. 2010; Anderson et al. 2011, 2013; Savolainen et al. 2013; Ågren et al. 2013, 2017; Bono et al. 2017). We predict that as beneficial mutations accrue in response to one selective challenge, their pleiotropic effects will generate trade‐offs observable in landscape patterns of phenotypic variation.

In this study, we examined whether functional trade‐offs develop as a result of the stepwise mutational changes that accumulate en route to an escalated adaptation. We tested whether alleles that confer tetrodotoxin (TTX) resistance in the common garter snake (*Thamnophis sirtalis*) also reduce other aspects of whole‐animal performance and underlying protein function. Populations of *T. sirtalis* in western North America evolved resistance to the neurotoxin TTX as a result of arms race coevolution with deadly prey, newts of the genus *Taricha* (Brodie et al. 2002; Hanifin et al. 2008). Throughout their sympatric range, garter snakes prey occasionally on newts along with other amphibians. Individual differences in snake resistance and newt toxicity predict whether a given predator‐prey interaction goes to completion, ending with the consumption of prey and/or incapacitation of predator (Williams et al. 2003, 2010). Population patterns of predator resistance and prey toxicity vary by several orders of magnitude across the spatial landscape, creating a geographic mosaic of coevolving traits with roughly matched abilities in both species. Snakes from two distinct coevolutionary “hotspots,” California and the Pacific Northwest, independently evolved resistance through convergent amino acid changes to the skeletal muscle voltage‐gated sodium channel (Na_V_1.4) that disrupt TTX‐binding at the molecular level (Fig. 1A; Geffeney et al. 2005; Hague et al. 2017).

**Figure 1:**
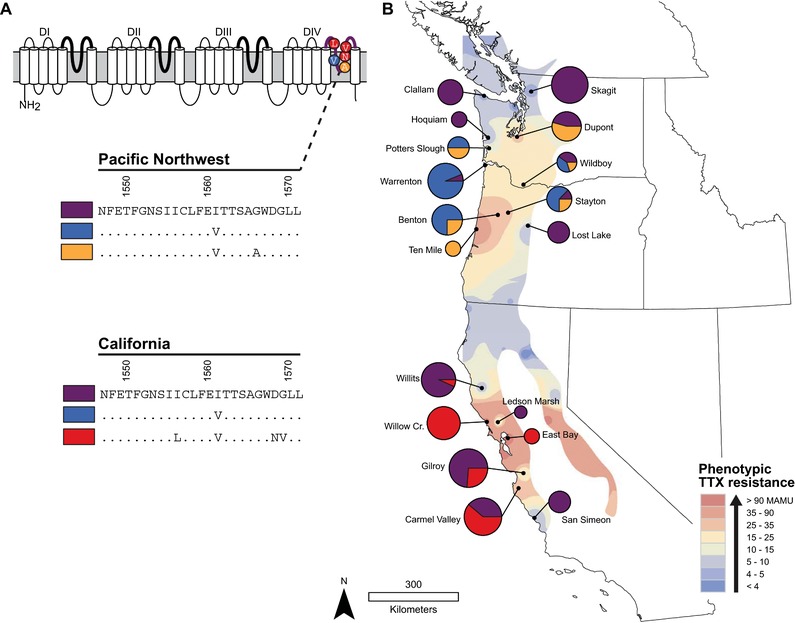
Substitutions in Na_V_1.4 arose independently in California and the Pacific Northwest. (A) Schematic of the Na_V_1.4 skeletal muscle sodium ion channel in *T. sirtalis*. Each domain (DI–DIV) is shown with the extracellular pore loops (p‐loops) highlighted with bold lines. Specific amino acid changes in the DIV p‐loop are show in their relative positions within the pore. Below, the TTX‐sensitive ancestral sequence (purple) is listed for each lineage of *T. sirtalis*, in California and the Pacific Northwest, followed by other alleles found in each region that are known to confer stepwise increases in channel resistance. (B) Pie charts indicate the frequencies of different homozygous neonates for each population sampled from the two lineages. Chart size is proportional to sample size. On the map background, population‐level average phenotypic TTX resistance (50% MAMU) of *T. sirtalis* is interpolated across the geographic range of sympatry with *Taricha* newts (figure adapted from Hague et al. 2017).

The deadly effects of TTX occur because the toxin binds to the outer pore of voltage‐gated sodium channels (Na_V_) in muscle and nerve tissue, blocking the influx of sodium ions and preventing action potential propagation (Fozzard and Lipkind 2010; Tikhonov and Zhorov 2012). The channels comprise four homologous domains (DI‐DIV), each of which contains a pore‐loop (p‐loop) that together form the outer pore of the channel where TTX molecules bind (Terlau et al. 1991; Fozzard and Lipkind 2010; Payandeh et al. 2011; Tikhonov and Zhorov 2012; Toledo et al. 2016). TTX‐resistant mutations to the DIV p‐loop of Na_V_1.4 arose in a stepwise fashion within each phylogenetically distinct lineage of *T. sirtalis* (Fig. 1A). An I1561V change (i.e., Na_V_1.4^V^) was the first resistant substitution to arise in both California and the Pacific Northwest, followed later by more resistant mutations to the DIV p‐loop that are unique within each lineage (Hague et al. 2017). Functional expression of derived alleles from California (Na_V_1.4^V^ and Na_V_1.4^LVNV^) and the Pacific Northwest (Na_V_1.4^V^ and Na_V_1.4^VA^) confirms they confer increasing levels of TTX resistance to Na_V_1.4 (Geffeney et al. 2005). Channel‐level TTX resistance conferred by each DIV allele is tightly correlated with muscle and whole‐animal levels of resistance (Geffeney et al. 2002, 2005; Feldman et al. 2010; Hague et al. 2017). Across western populations of *T. sirtalis*, TTX‐resistant alleles occur at high frequency within each of the two hotspots sympatric with toxic newts, but are largely absent in surrounding “coldspots” where newts are nontoxic (Brodie et al. 2002; Hanifin et al. 2008; Hague et al. 2017).

Within a few populations of garter snakes, it appears that TTX resistance is negatively correlated with locomotor performance—individuals with higher resistance crawl slower—suggesting a possible tradeoff associated with the evolution of resistance (Brodie and Brodie 1999). Crawl speed is an important measure of performance in reptiles (e.g., Shine et al. 2000; Aubret et al. 2007) and is under positive survival selection in some populations of garter snakes (Jayne and Bennett 1990). This relationship suggests that adaptation to toxic prey might come at a cost of reduced organismal performance, which also may be important in structuring broader mosaic patterns of coevolution (e.g., hotspots and coldspots). We investigated the underlying molecular basis for a putative trade‐off between TTX resistance and locomotor performance.

Some amino acid residues in the pore of Na_V_1.4 that determine TTX resistance also play a conserved role in electrical signaling in skeletal muscle tissue (Goldin 2002; Tikhonov and Zhorov 2005; Lee et al. 2011; Brodie and Brodie 2015; Toledo et al. 2016). Therefore, we predicted that mutations to the DIV p‐loop that disrupt toxin‐binding would generate a functional trade‐off between TTX resistance and other phenotypes related to muscle performance. Because resistance of the Na_V_1.4 channel evolved twice—in California and separately in the Pacific Northwest—we were able to conduct two evolutionarily independent tests for costs associated with TTX resistance. In each lineage, we tested for a relationship between DIV p‐loop genotype and phenotypic variation in crawl speed, a whole‐animal measure of muscle performance. Then, to evaluate the presumed underlying mechanism, we functionally expressed snake Na_V_1.4 channels in *Xenopus* oocytes and tested whether derived alleles in the DIV p‐loop caused pleiotropic changes to electrophysiological properties of the channel.

## Methods

### CRAWL SPEED ASSAY

If TTX‐resistant mutations to Na_V_1.4 also disrupt skeletal muscle function, then we expected snakes with derived genotypes in the DIV p‐loop to have reduced crawl speed compared to those with the ancestral, nonresistant channel. To test for a relationship between DIV genotype and crawl speed, we collected genotypic information from neonate snakes for which phenotypic variation in TTX resistance and crawl speed were previously collected (Brodie et al. 2002; Feldman et al. 2010; Hague et al. 2017). The final datasets included 77 neonate snakes from seven populations in California and 95 neonates from 11 populations in the Pacific Northwest at sites that co‐occur with *Taricha* newts (Fig. 1B, Table S1). These neonates were born in the lab to wild‐caught females, providing a uniformly aged sample of variation in crawl speed that was largely unexposed yet to postnatal selection.

Females were collected from the wild between 1985–2001 and 2004–2005 and returned to the laboratory at Utah State University. Within 24 hours of parturition, neonates were measured for mass (g), snout‐vent length (SVL; mm), and total length (mm), and then housed individually in 15 × 10.5 cm plastic tubs. Each neonate was stimulated to crawl for 2 m on a 4 × 0.1 m linear racetrack lined with indoor‐outdoor carpet. The racetrack was equipped with infrared sensors to electronically record sprint speed over 0.5 m intervals. Crawl speed was measured as the maximum velocity (m/s) over any 0.5 m interval. We raced each individual twice, and used the average as our crawl speed estimate. A single observer (EDB, Jr.) conducted all crawl speed trials in order to limit variance among observers. Previous work has shown that crawl speed estimates from this protocol are highly repeatable (Brodie and Brodie 1999; Brodie et al. 2002; Ridenhour et al. 2004; Feldman et al. 2010).

The neonates were also genotyped for their amino acid sequence in the DIV p‐loop of the Na_V_1.4 channel. Methods for DNA extraction from tail tip tissue and Sanger sequencing are described in Hague et al. 2017. For each individual, we sequenced a 666 bp fragment that includes the DIV p‐loop region of Na_V_1.4. Heterozygous positions on chromatograms were identified by eye and confirmed in both directions with sequencing. The haplotype phase of the DIV p‐loop sequence for each individual was inferred computational with the program PHASE (Stephens et al. 2001) and then translated into the amino acid sequence. We detected few subjects with a heterozygous DIV p‐loop, or from the California lineage with the Na_V_1.4^V/V^ genotype (see Table S1), so these individuals were removed from the dataset due to insufficient power.

We used R version 3.4.1 (R Core Team 2018) to test for a relationship between the response variable (neonate crawl speed [m/s]) and the independent variable, genotype of the DIV p‐loop in Na_V_1.4. We used a mixed modeling approach with the “lmer” function implemented in the *lme4* package (Bates et al. 2015). The DIV p‐loop genotype of each neonate was coded as a categorical fixed effect, with each unique genotype as a different level. We included SVL and mass in the model as fixed effects, because crawl speed in garter snakes scales with body length and mass (Arnold and Bennett 1988; Garland 1988; Brodie 1992, 1993). We also included the latitude of the population where each neonate originated in the wild as a fixed effect, because mean body size varies among populations in *T. sirtalis* (Brodie et al. 2002), and personal observations suggest that size varies along a latitudinal gradient. Finally, the population where each neonate originated was included as a random effect. Statistical significance of fixed effects was determined by an ANOVA using a Wald Chi‐Square test with type III sum of squares and one degree of freedom, implemented in the *car* R package (Fox and Weisberg 2011).

Our goal was to conduct a replicated test for trade‐offs with crawl speed in two monophyletic lineages of *T. sirtalis*, California and the Pacific Northwest, so we analyzed data from the two regions in separate statistical models. Populations in California and the Pacific Northwest are geographically separated and genetically divergent according to autosomal and mitochondrial loci (Janzen et al. 2002; Hague et al. 2017). Moreover, a gene tree of the Na_V_1.4 protein, based on genomic DNA from neonates in this study, indicates that TTX resistance in the DIV p‐loop evolved independently in the two lineages (Hague et al. 2017). Therefore, we assigned neonates to either the California or Pacific Northwest lineage based on the Na_V_1.4 tree. As a precaution, we did not include populations located in between the two lineages, along the California/Oregon border, because it is an apparent region of historical vicariance, and may now represent a contact zone between southern and northern lineages. Populations in this region all lack variation in Na_V_1.4, such that only the ancestral, nonresistant sequence (Na_V_1.4^+^) is found (Hague et al. 2017).

### HETEROLOGOUS EXPRESSION OF Na_V_1.4 Mutants

We tested whether changes in the biophysical function of the channel might underlie locomotor trade‐offs by evaluating the function of snake Na_V_1.4 channels expressed in heterologous *Xenopus* oocytes. We generated clones of Na_V_1.4 with the ancestral DIV p‐loop sequence (Na_V_1.4^+^) and two derived alleles (Na_V_1.4^V^ and Na_V_1.4^LVNV^), expressed each channel variant, and then measured TTX‐binding affinity (*K*_d_). We also recorded the voltage‐dependence of activation and fast‐inactivation (*V*_1/2_) in order to visualize channel excitability—the window current for which each channel is available to open and initiate action potentials in skeletal muscle tissue (Ketelaars et al. 2001; Remy et al. 2003; Barker et al. 2016).

The three different alleles were constructed in the background of a native, nonresistant Na_V_1.4 channel sampled from *T. sirtalis* in Illinois, outside the range of *Taricha* newts. Populations in Illinois are closely related and ancestral to western *T. sirtalis*, and contain the nonresistant p‐loop sequence of Na_V_1.4 (Janzen et al. 2002; Hague et al. 2017). All evidence suggests that western *T. sirtalis* and the Illinois sample share very high sequence similarity in Na_V_1.4 (99.7%) throughout the full 1875 amino acid sequence of the protein (Hague et al. 2017). Our Illinois construct improves upon previous expression work, which measured the effects of TTX‐resistant mutations from *T. sirtalis* in the divergent genetic background of mammalian Na_V_1.4 proteins (e.g., Geffeney et al. 2005; Lee et al. 2011). Due to resource constraints, we were only able to assess a limited number of mutants. We chose to focus our analysis on the two most common derived alleles in the wild (Na_V_1.4^V^ and Na_V_1.4^LVNV^).

The native Na_V_1.4 construct was generated using Gibson assembly (Gibson et al. 2009). We first used Sanger sequencing to generate the full protein‐coding sequence of Na_V_1.4 from an individual in Illinois (Hague et al. 2017). The synthetic Na_V_1.4 cDNA sequence (1875 aa, 5625 bp) was codon optimized (IDT) for expression in *Xenopus laevis* oocytes. Two silent EcoRV cut sites were included at positions 4482 and 5211 to allow for mutagenesis. We used a commercial supplier (IDT) to generate four synthetic oligonucleotides (≈1400 bp each) that corresponded to the codon‐optimized cDNA. The blocks included 20 bp overlapping regions with each other and the target vector to enable Gibson assembly. We assembled gene fragments with a linearized (SmaI, NEB) vector (pGEMHE, courtesy of J. Rosenthal) that included a T7 promotor for in vitro mRNA synthesis, 3’ and 5’ *Xenopus* globin UTRs, and a poly‐A tail using standard Gibson assembly protocols (NEB). The product of this reaction was transformed into competent JM109 cells (Promega, USA) and selectively screened using standard protocols. Positive clones were sequenced using Sanger sequencing (Sequetech; USA) to ensure correct assembly and orientation of the Na_V_1.4 sequence. We chose one correct clone, which was retransformed and sequence verified for further expression and mutagenesis.

The three channel variants were then constructed using Gibson assembly. Sequence‐verified plasmids with the complete Na_V_1.4 insert were digested with EcoRV (NEB) and purified in agarose gel (0.8%) to isolate the 8.5 kb fragment. The fragment was further purified and concentrated using standard Phenol:Choloroform protocols and Na^+^ acetate precipitation. The resulting linearized plasmid was identical to the native Na_V_1.4 construct with approximately 700 bp removed from the DIV region of the protein. We constructed all three DIV alleles (Na_V_1.4^+^, Na_V_1.4^V^, and Na_V_1.4^LVNV^) with the same approach. The three different constructs were then linearized with a Nhe1 digestion (NEB). We used a T7 ultra mMessage mMachine kit (Life Technologies) to synthesize capped and tailed mRNAs and then injected 5–30 ng of each channel clone mRNA into stage 5 *Xenopus* oocytes (EcoCyte Bioscience).

Ionic currents were measured at room temperature (22–25°C) 2–7 days after mRNA injection using the cut‐open oocyte Vaseline gap voltage‐clamp technique (Stefani and Bezanilla 1998) with a CA‐1B High Performance Oocyte Clamp (Dagan Instruments). Recordings were made in an external solution containing (in mM): 120 Na‐Mes, 10 Hepes‐Na, 1.8 CaCl_2_, pH 7.2 and an internal solution containing (in mM): 110 K‐Mes, 10 Na‐Mes, 10 Hepes‐K, 1 EGTA‐K, pH 7.2. Current records were acquired using Axon pClamp software (version 10, Molecular Devices), sampling at 100 kHz and filtering at 20 kHz. The holding potential for all experiments was –100 mV. Leak subtraction was performed with the use of a *p*/4 protocol.

We first measured TTX‐binding affinity to assess the TTX resistance of each channel clone. Peak currents were evoked at 0.05 Hz with 20‐ms pulses to 0 mV following a 500 ms prepulse to –150 mV. Peak current amplitudes were measured offline with Igor Pro (Wavemetrics). The ratio of peak currents in the presence and absence of TTX over a range of TTX concentrations were calculated with peak currents recorded before and after perfusing the selected TTX concentration into the external bath solution for 5 minutes. To estimate the TTX concentration that blocked 50% of the expressed channels, the data were fitted to an equation derived from a single‐site Langmuir adsorption isotherm, current ratio = 1/1+[TTX]/*K*_d_ in which [TTX] is the concentration of toxin and *K*_d_ is the concentration of TTX at which half of the channels are bound to the toxin. *K*_d_ and its 95% confidence limits were estimated from the curve using Igor Pro (Wavemetrics).

We next measured the voltage‐dependence of activation and fast‐inactivation to assess the gating properties of each cloned channel. The voltage‐dependence of activation was measured from the peak inward current during a 20 ms test pulse to voltages ranging from –100 to 80 mV in 10 mV steps following a 500‐ms prepulse to –150 mV. The voltage‐dependence of fast‐inactivation was measured from the peak inward current during a 20 ms pulse to 0 mV after a 500 ms, conditioning prepulse ranging from –150 to –10 mV in 10 mV increments. Peak current amplitudes were measured during test pulses offline with Igor Pro (Wavemetrics). Conductance‐voltage relationships were derived using the following equation: *G*_Na_ = *I*_peak_/(*V*_M_ – *E*_Na_) where *G*_Na_ represents sodium conductance, *I*_peak_ is the peak‐test‐pulse current, *V*_M_ is the test‐pulse voltage, and *E*_Na_ is the measured sodium equilibrium potential. Activation and fast‐inactivation curves were fitted by a Boltzmann distribution with the following equation: Normalized conductance or current amplitude = 1/(1+ exp(‐*ze*_0_(*V*_M_ – *V*_1/2_)/*kT*)) where *z* is the apparent valence, *e*_0_ is the elementary charge, *V*_1/2_ is the midpoint voltage, *k* is the Boltzmann constant, and *T* is the temperature in degrees Kelvin. *V*_1/2_ and its 95% confidence limits were estimated from the curve using Igor Pro (Wavemetrics). Finally, for each cloned variant, we visualized channel window current as the area below the normalized overlapping activation and fast‐inactivation curves.

## Results and Discussion

### RESISTANCE MUTATIONS ARE LINKED TO REDUCED CRAWL SPEED

**Figure 2:**
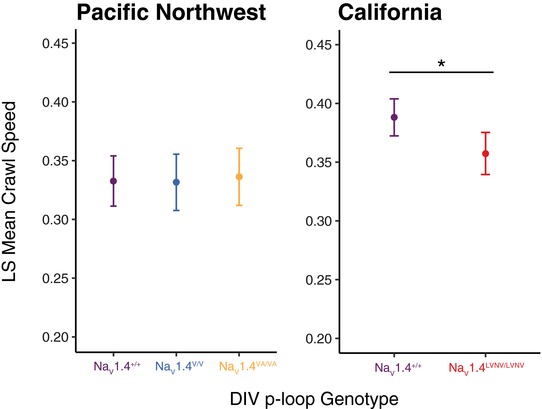
Neonates from California with a TTX‐resistant genotype show reductions in crawl speed. Least square (LS) mean velocity (± 95% CI) of neonates with different homozygous DIV genotypes (colors as in Fig. 1). LS means for the Pacific Northwest and California datasets were derived from separate LMMs. Na_V_1.4^V/V^ homozygotes from California and all heterozygotes were not included in the analyses because they were so rare (see Table S1).

In the Pacific Northwest lineage, we did not find a significant relationship between DIV p‐loop genotype and crawl speed. Body mass was the only significant fixed effect in the model (Table 1; Wald χ^2^ = 4.02, *P* = 0.045), which is consistent with previous work that shows locomotor ability depends on mass and SVL in *Thamnophis* species (Arnold and Bennett 1988; Garland 1988; Brodie 1992, 1993). Unlike in the Pacific Northwest, in California we found that the DIV p‐loop genotype accounted for significant variance in crawl speed of neonate snakes (Wald χ^2^ = 6.09, *P* = 0.014). Animals with the highly TTX‐resistant Na_V_1.4^LVNV/LVNV^ genotype had a slower mean crawl speed than individuals with the ancestral wild‐type channel (Fig. 2). SVL also significantly affected crawl speed in the California lineage (Wald χ^2^ = 16.10, *P* < 0.001).

**Table 1:** Results of linear mixed models (LMMs) testing effects on crawl speed for each garter snake lineage

	Pacific Northwest		California	
Fixed‐effect	Wald χ^2^	*P*‐value	Wald χ^2^	*P*‐value
DIV p‐loop genotype	0.16	0.924	6.09	0.014^*^
SVL	3.66	0.056	16.1	0.000^*^
Mass	4.02	0.045^*^	0.01	0.92
Latitude	0.51	0.477	0.64	0.423

The pattern observed in the California lineage suggests a compromise between two phenotypes linked to the function of Na_V_1.4 in skeletal muscle tissue: TTX resistance and crawl speed. The four amino acid substitutions in the Na_V_1.4^LVNV^ allele, shown previously to confer large increases in whole‐animal resistance (Geffeney et al. 2002, 2005; Feldman et al. 2010; Hague et al. 2017), appear to disrupt muscle performance to an extent that is detectable at the organismal level. It is unlikely the reduction in crawl speed is due to unaccounted for changes in other regions of Na_V_1.4 linked to the DIV p‐loop, because the majority of protein‐coding sequence is extremely conserved in *T. sirtalis*. Of the 1875 residues in the Na_V_1.4 channel, only one other amino acid position in western *T. sirtalis* exhibits polymorphism outside of the substitutions in the DIV p‐loop examined here. That single change is distantly located in the intracellular portion of the protein, such that it is unlikely to influence channel biophysics or occur in linkage with substitutions in the DIV p‐loop (Hague et al. 2017).

The changes found in the Na_V_1.4^LVNV^ allele represent a late escalatory stage of TTX resistance in the arms race with toxic newts. Of all derived alleles in either California and the Pacific Northwest, Na_V_1.4^LVNV^ contains the most amino acid changes to the channel pore and confers the largest increase in phenotypic TTX resistance (Hanifin et al. 2008; Hague et al. 2017). The Na_V_1.4^LVNV^ channel is an order of magnitude more resistant than any other known variant in *T. sirtalis* (see below; Geffeney et al. 2005), and snakes with even one copy of Na_V_1.4^LVNV^ have extremely high levels of phenotypic TTX resistance (Feldman et al. 2010). In fact, *T. sirtalis* in the California lineage are so resistant they appear to have escaped the arms race and can consume sympatric newts with little or no consequence (Hanifin et al. 2008). This level of escalation has not occurred in the Pacific Northwest lineage. Reduced crawl speed in California, but not in less‐resistant populations from the Pacific Northwest, implies that negative trade‐offs only arise late in the adaptive trajectory of the TTX‐resistant Na_V_1.4 channel.

We tested for categorical differences in crawl speed among DIV p‐loop genotypes, but previous work suggests a trade‐off between whole‐animal TTX resistance and crawl speed may also occur on a continuous scale at the individual level. In populations from the Pacific Northwest, Brodie and Brodie (1999) found that individual variation in phenotypic TTX resistance was negatively associated with crawl speed (although the DIV genotype of each individual was unknown). Our model did not find evidence for a trade‐off in the Pacific Northwest; however, mutations to the pore of Na_V_1.4 are not the sole determinant of whole‐animal TTX resistance (McGlothlin et al. 2014, 2016; Feldman et al. 2016). Consequently, there may be other unknown mechanisms that contribute to a trade‐off between physiological resistance and crawl speed.

### RESISTANCE MUTATIONS ALTER CHANNEL FUNCTION

**Figure 3:**
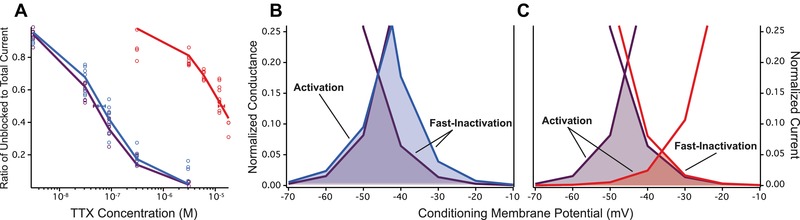
TTX‐resistant alleles change functional measures of Na_V_1.4 channel activity. (A) TTX resistance of three cloned Na_V_1.4 channels from *T. sirtalis*. Each channel is color‐coded according to its DIV sequence in Figure 1. The TTX concentration that blocked 50% of the channels (*K*_d_) for each channel type was calculated from pooled channel data. Lines represent the equations fitted to the data for each channel and *K*_d_ values (± 95% CI) are shown with a horizontal bar. Next, the window currents for the (B) Na_V_1.4^V^ and (C) Na_V_1.4^LVNV^ channels are shown as the shaded area below the normalized overlapping activation and fast‐inactivation curves. Each channel is shown in comparison to the ancestral Na_V_1.4^+^ channel (in purple). The voltage‐dependence of activation and fast‐inactivation (including *V*_1/2_ ± 95% CI) were measured by fitting the data with a Boltzmann function (see Fig. S1).

Heterologous expression of cloned Na_V_1.4 variants demonstrated the Na_V_1.4^V^ channel had a small increase in TTX resistance compared to the ancestral wild‐type (*K*_d_ = 65 nM; Fig. 3A), which was coupled with a 7 mV shift in the voltage‐dependence of fast‐inactivation toward more depolarized potentials (*V*_1/2_ = −49.2 mV; Table 2, Fig. S1). These changes resulted in a slight overall increase in the window current of the channel (Fig. 3B). The Na_V_1.4^LVNV^ channel, in contrast, generated a dramatic 260‐fold increase in TTX resistance (*K*_d_ = 13000 nM; Fig. 3A) coupled with a large 20 mV shift in the voltage‐dependence of activation toward more depolarized potentials (*V*_1/2_ = –16.4 mV; Table 2, Fig. S1). The depolarized shift in activation led to a clear reduction in the window current of the Na_V_1.4^LVNV^ channel (Fig. 3C).

**Table 2:** TTX resistance and channel function as measured on cut‐open voltage clamp recording

		TTX resistance		Activation		Fast‐inactivation
Na_V_1.4 mutant	*n*	*K*_d_ ± CI (nM)	*n*	*V*_1/2_ ± CI (mV)	*n*	*V*_1/2_ ± CI (mV)
Na_V_1.4^+^	13	50 ± 5.2	7	–36.2 ± 1.0	9	−56.6 ± 0.7
Na_V_1.4^V^	11	65 ± 11	8	–34.7 ± 1.9	8	−49.2 ± 0.8
Na_V_1.4^LVNV^	11	13000 ± 1800	7	–16.4 ± 0.5	10	−54.7 ± 0.7

For each channel type, TTX resistance was measured as the TTX concentration that blocked 50% of channels (*K*_d_ ± 95% CI). The voltage values (mV) are shown for which 50% of channels are open due to activation and closed due to fast‐inactivation (*V*_1/2_ ± 95% CI).

Our results indicate that TTX‐resistant mutations to the channel pore have pleiotropic effects on important aspects of protein function. The large shifts in voltage‐dependence of activation and window current found in the Na_V_1.4^LVNV^ clone suggest that TTX‐resistant mutations cause a reduction in the excitability of Na_V_1.4 channels in skeletal muscle tissue. These shifts were not observed in past experiments that expressed the same TTX‐resistant substitutions in the foreign genetic background of a mammalian Na_V_1.4 channel (Lee et al. 2011). Mutations to the DIV p‐loop disrupt toxin‐binding at the outer pore, but they also occur in an important region for gating and ion conductance in voltage‐gated (Na_V_) sodium channels (Vilin and Ruben 2001; Hilber et al. 2005; Xiong et al. 2006; Lee et al. 2011). The p‐loop sequences are otherwise highly conserved in vertebrates (Goldin 2002; Tikhonov and Zhorov 2005; Brodie and Brodie 2015; Toledo et al. 2016; Hague et al. 2017) and Na_V_1.4 is thought to be under strong purifying selection for the maintenance of its important role in electrical signaling of muscle tissue (Brodie and Brodie 2015). The changes to excitability we observed in the Na_V_1.4^LVNV^ clone are consistent with a trade‐off between TTX resistance and muscle performance in the crawl speed assay. Similar depolarizing shifts in the voltage‐dependence of activation, for example, occur in humans with a congenital myopathy that causes general muscle weakness and delays in developmental milestones like walking (Zaharieva et al. 2016). A number of mutations to Na_V_1.4 in humans are linked to comparable muscle pathologies like paralysis and weakness (Cannon 1996; Lehmann‐Horn and Jurkat‐Rott 1999; Vilin and Ruben 2001; Jurkat‐Rott et al. 2015; Nicole and Fontaine 2015; Zaharieva et al. 2016; Hinard et al. 2017).

Ultimately, evolution of the pore sequence of Na_V_1.4 must strike a balance between TTX‐resistant properties and the maintenance of channel function (Feldman et al. 2012; Brodie and Brodie 2015; Toledo et al. 2016). Our results are consistent with other work that shows TTX‐resistant mutations in the DIV p‐loop affect a range of biophysical properties in Na_V_ channels. Slow‐inactivation, a more prolonged form of Na_V_ inactivation, is also altered by changes to the pore of the channel. TTX‐resistant mutations in the Na_V_1.4^LVNV^ allele have been shown to alter the voltage‐dependence of slow‐inactivation toward more depolarized membrane potentials (Lee et al. 2011; Toledo et al. unpubl. data). In addition to gating, amino acid residues in the pore are critically responsible for the selective influx of Na^+^ ions that propagate action potentials. TTX‐resistant mutations to the pore can disrupt Na^+^ conductance (Terlau et al. 1991; Feldman et al. 2012) and increase calcium ion permeability (Heinemann et al. 1992). For example, the D1568N amino acid substitution in Na_V_1.4^LVNV^ removes a negative charge that interacts with TTX, but also causes a decrease in ion conductance (Terlau et al. 1991; Toledo et al. 2016).

TTX‐resistant mutations in the California lineage clearly affect important electrophysiological properties of Na_V_1.4 and correlate with reductions in organismal performance of crawl speed. However, the mechanistic link between changes to Na_V_1.4 function and reduced organismal performance still remains untested. To unequivocally demonstrate a functional link between TTX‐resistant mutations, their electrophysiological effects, and locomotor performance would require direct recordings from muscle fibers of snakes with known genotypes. Only then could we establish whether the reduced excitability we observed in Na_V_1.4^LVNV^ causes changes to threshold and speed of action potential conductance in skeletal muscle tissue. Therefore, we cannot rule out alternative explanations for the relationships we detected between DIV genotype, channel function, and organismal performance. For example, compensatory effects in the muscle cells of TTX‐resistant snakes, like changes to Na_V_1.4 expression or the sodium‐potassium pump, could ameliorate reduced excitability in Na_V_1.4^LVNV^. In addition, the four mutations in the DIV p‐loop of Na_V_1.4^LVNV^ might have different functional consequences depending on their genetic background. We inserted the DIV allele into an Na_V_1.4 background based on an Illinois snake, which differs in sequence identity from western populations by no more than five other amino acids substitutions. However, the sequences are otherwise 99.7% identical throughout the 1875 amino acid positions of the channel (Hague et al. 2017), and the five differences occur in regions that do not regulate activation or fast‐inactivation. Thus, we consider it unlikely that this small number of differences would dramatically confound our interpretations.

### CONCLUSION

As a population evolves toward a new adaptive peak, phenotypic compromises are expected to arise if an underlying allele impacts multiple aspects of organismal performance (Felsenstein 1976; Hedrick et al. 1976; Hedrick 1986, 2006; Kawecki and Ebert 2004; Bono et al. 2017). In the arms race with toxic newts, populations of *T. sirtalis* that evolved exaggerated TTX resistance experience an apparent trade‐off as mutations accumulate in the otherwise conserved pore region of Na_V_1.4. These costs are not clear at every mutational step, such as the single substitution we examined from the Pacific Northwest, but they become evident where coevolution has led to extreme phenotypes and the largest number of substitutions at the underlying level. The trade‐off we observed may ultimately limit coevolutionary dynamics if snakes experience a fitness cost. Garter snakes must avoid their own predators, including birds and mammals, and crawl speed in *T. sirtalis* has previously been shown to influence survival (Jayne and Bennett 1990; Shine et al. 2000). A phenotypic trade‐off between resistance and locomotion has important implications for landscape‐level patterns of coevolution. For example, TTX‐resistant alleles like Na_V_1.4^LVNV^ may be favored in localities where toxic newts represent strong reciprocal selection, but disfavored in areas with nontoxic newts where reduced crawl speed and antipredator ability are more important contributors to survival.

Geographic patterns of Na_V_1.4 polymorphism appear to support balancing selection for such a trade‐off in the arms race. In wild populations, TTX‐resistant alleles occur at high frequency in geographic “hotspots” with toxic newts, but at low frequency in neighboring “coldspots” where newts have little or no toxin (Hanifin et al. 2008; Hague et al. 2017). This mosaic pattern implies the existence of spatial variation in selection on Na_V_1.4 alleles. In California, allele frequencies shift from predominantly TTX‐resistant (Na_V_1.4^LVNV^) to nonresistant (Na_V_1.4^+^) over the short geographic distance of about 150 km (Hague et al. 2017). For alleles with pleiotropic effects, like Na_V_1.4^LVNV^, balancing selection is expected to maintain genetic polymorphism across a heterogeneous landscape of selection, like a mosaic of variably toxic newt populations (Turelli and Barton 2004; Charlesworth 2006; Mitchell‐Olds et al. 2007). At conserved loci of large effect, like the Na_V_1.4 channel, maintenance of polymorphism may be predicted because single mutations result in trade‐offs that alter whole‐animal measures of performance.

## DATA ACCESSIBILITY

DNA sequence alignments of the DIV p‐loop and the Na_V_1.4^+^ sequence from Illinois are deposited in GenBank (KY744954‐KY745723 and MH316124, respectively). Crawl speed and heterologous expression data will be submitted to Dryad upon manuscript acceptance.

Associate Editor: K. Lythgoe

## Supporting information


**Figure S1**. Estimated curves for the voltage‐dependence of activation and fast‐inactivation.
**Table S1**. Sampling information for populations from the California and Pacific Norwest datasets.
