# Supplementary material for: Large‐effect mutations generate trade‐off between predatory and locomotor ability during arms race coevolution with deadly prey
Source: Evol Lett. 2018 Jul 31;2(4):406–16. doi: 10.1002/evl3.76 (PMC6121790; doi:10.1002/evl3.76)
Supplement: Supplementary file 1 — Figure S1. Estimated curves for the voltage‐dependence of activation and fast‐inactivation. Table S1. Sampling information for populations from the California and Pacific Norwest datasets. [file EVL3-2-406-s001.docx]

**SUPPLEMENTAL INFORMATION**

**
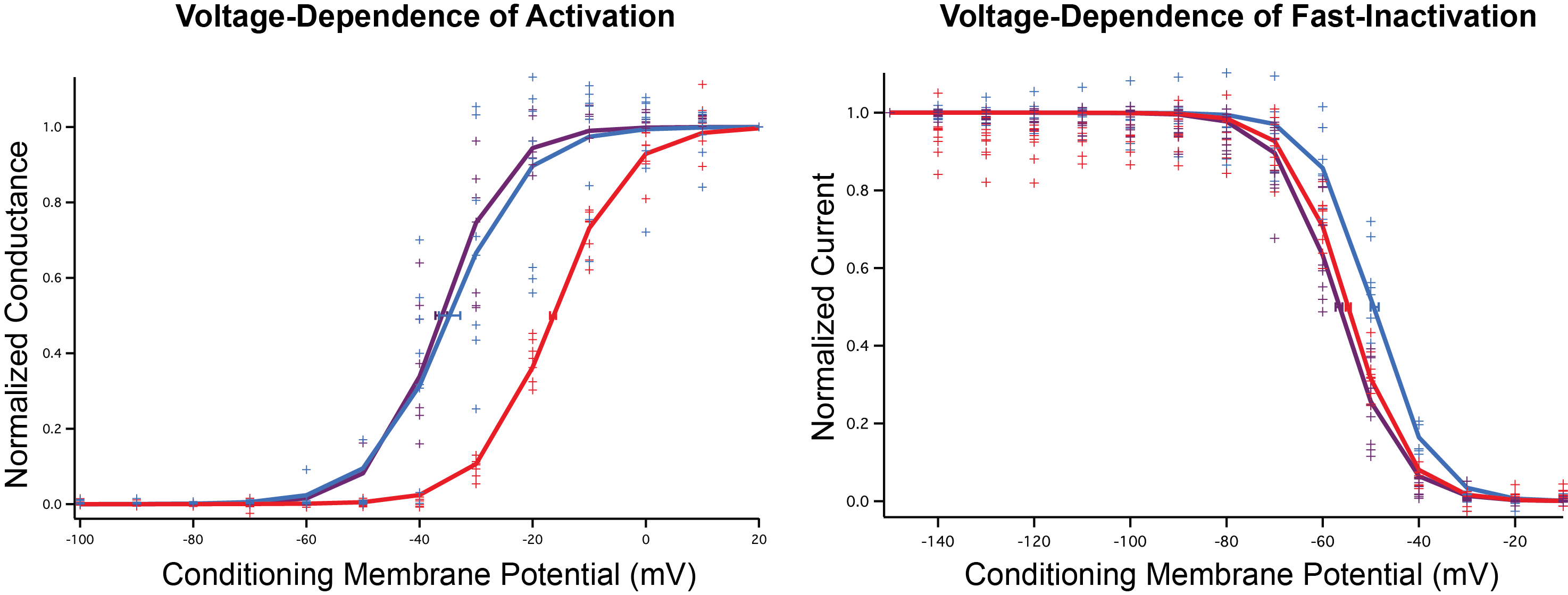
**

**Figure S1 related to Figure 3.** Estimated curves for the voltage-dependence of activation and fast-inactivation. Each mutant channel is color-coded according to its DIV sequence in Figure 1 (i.e. purple = Na_V_1.4^+^, etc.). The membrane voltage at which 50% of the channels are activated or inactivated (*V*_1/2_) for each channel was calculated by fitting pooled channel data with a Boltzmann function. *V*_1/2_ values (± 95% C.I.) are shown with a horizontal bar.

**Table S1 related to Figure 1.** Sampling information for populations from the California and Pacific Norwest datasets. Individual sample sizes are show for each DIV p-loop genotype. Na_V_1.4^V/V^ homozygotes from California and all heterozygotes were removed from the analyses due to low sample sizes and a lack of power.

| California lineage | |  |  |  |  |  |  |  |
| --- | --- | --- | --- | --- | --- | --- | --- | --- |
| Population | **County** | **Latitude** | **Longitude** | **Na_V_1.4^+/+^** | **Na_V_1.4^V/+^** | **Na_V_1.4^V/V^** | **Na_V_1.4^LVNV/+^** | **Na_V_1.4^LVNV/LVNV^** |
| Carmel Valley | Monterey, CA | 36.482112 | -121.717191 | 7 | - | - | 2 | 11 |
| East Bay | Contra Costa, CA | 37.977521 | -122.228124 | - | - | - | - | 3 |
| Gilroy | Santa Cruz, CA | 36.946163 | -121.563676 | 14 | 1 | 2 | 9 | 5 |
| Ledson Marsh | Sonoma, CA | 38.446913 | -122.647488 | 2 | 4 | 1 | - | - |
| San Simeon | San Luis Obispo, CA | 35.603385 | -121.079395 | 6 | - | 1 | - | - |
| Willits | Mendocino, CA | 39.44217 | -123.35263 | 14 | - | - | 1 | 1 |
| Willow Cr. | Sonoma, CA | 38.431705 | -123.06943 | 0 | - | - | - | 14 |
|  |  |  | **Total** | **43** | **5** | **4** | **12** | **34** |

| Pacific Northwest lineage | |  |  |  |  |  |  |  |  |
| --- | --- | --- | --- | --- | --- | --- | --- | --- | --- |
| Population | **County** | **Latitude** | **Longitude** | **Na_V_1.4^+/+^** | **Na_V_1.4^V/+^** | **Na_V_1.4^V/V^** | **Na_V_1.4^VA/V^** | **Na_V_1.4^VA/+^** | **Na_V_1.4^VA/VA^** |
| Benton | Benton., OR | 44.699936 | -123.221014 | - | - | 9 | 6 | - | 3 |
| Clallam | Clallam, WA | 48.25144 | -124.26268 | 8 | - | - | - | - | - |
| Dupont | Pierce, WA | 47.0972 | -122.63454 | 5 | - | - | - | 3 | 6 |
| Hoquiam | Grays Harbor, WA | 47.009425 | -123.911207 | 3 | - | - | - | - | - |
| Lost Lake | Hood River, OR | 44.442778 | -121.918611 | 6 | - | - | - | - | - |
| Potters Slough | Pacific, WA | 46.68519 | -123.82902 | - | 1 | 3 | 2 | 1 | 3 |
| Skagit | Skagit, WA | 48.49134 | -122.15052 | 17 | - | - | - | - | - |
| Stayton | Marion, OR | 44.792182 | -122.794489 | 1 | - | 5 | 1 | - | 2 |
| Ten Mile | Lane, OR | 44.222224 | -124.075364 | - | - | - | - | - | 3 |
| Warrenton | Clatsop, OR | 46.16807 | -123.93873 | 1 | - | 15 | 1 | - | - |
| Wildboy | Skaminia, WA | 45.671883 | -122.216926 | 2 | - | 2 | 1 | - | 1 |
|  |  |  | **Total** | **43** | **1** | **34** | **11** | **4** | **18** |
